# Causal Effects of Inflammatory Cytokines on Bipolar Disorder: A Bidirectional Two‐Sample Mendelian Randomization Study

**DOI:** 10.1002/brb3.70796

**Published:** 2025-09-07

**Authors:** Ying Cheng, Meiti Wang, Yu Fang, Jinjie Xu, Jinxin Zheng, Peijun Ju, Jianhua Chen

**Affiliations:** ^1^ Shanghai Mental Health Center Shanghai Jiao Tong University School of Medicine Shanghai China; ^2^ Beijing Anding Hospital Capital Medical University Beijing China; ^3^ Department of Nephrology, Ruijin Hospital, Institute of Nephrology Shanghai Jiao Tong University School of Medicine Shanghai China; ^4^ School of Global Health, Chinese Center for Tropical Diseases Research Shanghai Jiao Tong University School of Medicine Shanghai China; ^5^ Shanghai Key Laboratory of Psychotic Disorders Shanghai China; ^6^ Shanghai Institute of Traditional Chinese Medicine for Mental Health Shanghai China; ^7^ Yueyang Hospital of Integrated Chinese and Western Medicine Shanghai University of Traditional Chinese Medicine Shanghai China; ^8^ The First Affiliated Hospital of Xinjiang Medical University Xinjiang China

**Keywords:** bipolar disorder, causality, cytokines, Mendelian randomization

## Abstract

**Introduction:**

Inflammatory cytokine disturbance is a prominent outcome of immune dysregulation, extensively documented in bipolar disorder (BD). However, observational studies have exhibited inconsistent findings, and the causal relationships between inflammatory factors and BD remain unclear. Hence, this study aimed to uncover the causality between circulating inflammatory cytokines and BD.

**Methods:**

In the bidirectional Mendelian randomization (MR) analysis, two genetic instruments derived from a publicly available genomic dataset were utilized. Genetic variant data for 41 inflammatory cytokines were obtained from a meta‐analysis of genome‐wide association studies involving 8293 Finnish individuals. BD data included 41,917 cases and 371,549 controls from the Psychiatric Genomics Consortium Database. To estimate causal connections between inflammation cytokines and BD, we performed five methods: inverse variance weighting (IVW), MR‐Egger, weighted median, simple mode, and weighted mode. Sensitivity analyses were conducted to evaluate robustness, including tests for heterogeneity, pleiotropy, and leave‐one‐out validation.

**Results:**

In the IVW approach, we identified significant associations between genetic liability to elevated levels of interleukin‐17 (IL‐17), macrophage inflammatory protein‐1α (MIP‐1α), and monocyte chemotactic protein 3 (MCP‐3) with increased risk of developing BD (odds ratio [OR] = 1.119, 95% confidence interval [CI] = 1.021–1.226, *p* = 0.016; OR = 1.084, 95% CI = 1.002–1.174, *p* = 0.044; OR = 1.060, 95% CI = 1.001–1.122, *p* = 0.046, respectively). Across the various MR methods employed, the directions of causal inferences remained consistent. However, reverse MR analysis revealed no significant evidence for causal effects of genetic predisposition to BD on inflammatory cytokines.

**Conclusion:**

Our findings provide robust genetic evidence supporting causal effects of elevated circulating inflammatory cytokines on BD, conferring a high susceptibility to BD. Our study emphasizes that interventions aimed at reducing peripheral inflammatory cytokine levels should be prioritized in BD management.

## Graphical abstract

This study utilized a bidirectional Mendelian randomization approach to delineate the causal relationships between inflammatory cytokines and bipolar disorder. The analysis identified IL‐17, MIP‐1α, and MCP‐3 as being causally linked to an increased risk of bipolar disorder, highlighting their potential roles in the disease etiology and offering promising targets for future therapeutic strategies.

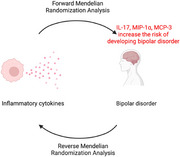

## 1 Introduction

Bipolar disorder (BD) is a prevalent and debilitating mood disorder, encompassing bipolar I and bipolar II subtypes. The lifetime prevalence of BD worldwide exceeds 1%, characterized by recurrent manic, hypomanic, and depressive episodes (McIntyre et al. 2020). BD patients have a significantly increased risk of suicide, with suicide attempts reaching 60% and suicide deaths affecting up to 20% of patients, imposing substantial socioeconomic burdens and wide‐ranging consequences (Dembek et al. 2023). The heritability of BD is estimated to be 70%, underpinned by a polygenic architecture, with genome‐wide association studies (GWAS) uncovering a great deal of common genetic markers (Mullins et al. 2021). Furthermore, emerging evidence highlights immune and inflammatory dysfunction—particularly “low‐grade inflammation”—as a key pathophysiological factor in BD (Poletti et al. 2024). For instance, BD patients show increased susceptibility to autoimmune diseases (Pape et al. 2019), T‐cell abnormalities (Z. Chen et al. 2023), and altered levels of pro‐inflammatory cytokines and chemokines (Poletti et al. 2024). Elevated peripheral inflammatory markers like interleukin‐6 (IL‐6) and tumor necrosis factor‐α (TNF‐α) are correlated with structural and functional brain abnormalities in BD patients (Saccaro et al. 2023). Notably, lithium, as a mood stabilizer with anti‐suicidal effects, and anti‐inflammatory drugs reduce inflammatory factors and improve clinical mood symptoms in BD subjects (Ruiz‐Sastre et al. 2024). Despite these findings, the potential causal relationship between immune‐inflammatory factors and BD remains unclear due to limitations of observational studies, such as small sample size, population ancestry differences, limited statistical methods, and confounding factors.

Nowadays, existing observational evidence alone cannot resolve the causal direction between circulating cytokines and BD: whether cytokines drive BD risk, or does BD influence cytokines. Mendelian randomization (MR) analysis, which leverages genetic variants as instrumental variables (IVs), typically represented by single‐nucleotide polymorphisms (SNPs), provides a robust approach for inferring causal relationships free from traditional epidemiological biases (Sanderson et al. 2022). In this study, we conducted a bidirectional two‐sample MR analysis using publicly available GWAS data to assess the causal effects of inflammatory cytokines on BD and the reverse associations from BD to cytokine.

## 2 Materials and Methods

### 2.1 Study Design Overview

**FIGURE 1:**
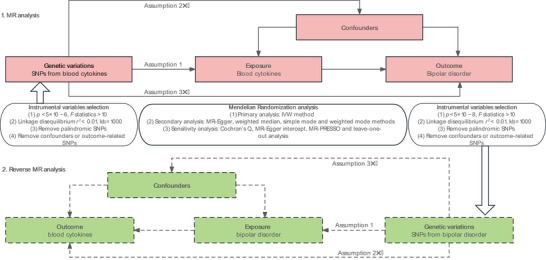
The flowchart of Mendelian randomization in the study. Assumption 1: Genetic variations are strongly associated with the exposure; Assumption 2: Genetic variations are not related to confounding factors from the exposure–outcome; Assumption 3: Genetic variations affect outcomes only through the selected exposure and not through any other pathway. SNPs, single nucleotide polymorphisms; MR, Mendelian randomization; MR‐PRESSO, Mendelian randomization pleiotropy residual sum and outlier.

The study flowchart is presented in Figure 1. We performed a bidirectional two‐sample MR analysis to investigate the causal relationships between 41 peripheral immune cytokines and BD utilizing summary statistics from European‐ancestry GWAS. First, we examined the causal effects of immune cytokines on BD by selecting genetic variants associated with each cytokine as IVs. Second, we reversed the analysis to explore potential causal effects of BD on these cytokines. The MR design adhered to three core assumptions: (1) the selected genetic IVs were strongly correlated with the exposure; (2) the IVs were not associated with any confounding factors; (3) the selected genetic variations influenced the outcome solely through the exposure (L.G. Chen et al. 2024).

### 2.2 GWAS Summary‐Level Data for Circulating Cytokines

The GWAS summary statistics for 48 blood inflammatory cytokines were obtained from the Young Finns Study (YFS), and two FINRISK cohorts (1997 and 2002) (Ahola‐Olli et al. 2017). These studies collectively included 8293 Finnish individuals aged 25–74 years with cardiovascular risk factors. Cytokine levels were quantified using ELISA across three cohorts: YFS, FINRISK 1997, and FINRISK 2002. Subjects with measurements outside detectable assay limits were excluded. Cytokines exhibiting > 90% missing data rates (7 of 48 initially measured) were also excluded. Following quality control, 41 cytokines were retained in the present study. Thus, the GWAS data from these sources were available at https://www.ebi.ac.uk/gwas/home.

### 2.3 GWAS Summary‐Level Data for Bipolar Disorder

The GWAS summary statistics for BD were obtained from the Psychiatric Genomics Consortium website (https://www.med.unc.edu/pgc/results‐and‐downloads; Mullins et al. 2021). This particular GWAS comprised a total of 41,917 BD patients and 371,549 healthy controls of European ancestry. The analysis identified 64 significant genomic loci, including 33 novel risk loci not previously associated with BD, that surpassed the threshold of *p* < 5 × 10^−8^.

### 2.4 Instrumental Variables Selection

In the analysis of blood cytokines, since only a limited number of cytokines had more than two independent SNPs at genome‐wide significance levels with *p* < 5 × 10^−8^ (linkage disequilibrium *r* ^2^< 0.01, kb = 1000). We adopted an alternative threshold of *p* < 5 × 10^−6^ to include more SNPs as IVs, which has been previously suggested (Burgess et al. 2013; J. Zhang, Li, et al. 2024) (Table S1). For BD, we adopted a significance level of *p* < 5×10^−8^. We utilized Phenoscanner (www.Phenoscanner.medschl.cam.ac.uk), removing SNPs associated with known BD risk factors or inflammatory mediators, to mitigate potential confounding factors. For the cytokine GWAS summary data, we systematically excluded SNPs associated with established BD risk factors, such as other mental disorders (major depression, schizophrenia, personality disorder, etc; Cross‐Disorder Group of the Psychiatric Genomics Consortium 2019), alcohol consumption (G. Li et al. 2024), smoking status, and sleep quality (Firth et al. 2020). In regard to GWAS summary data for BD, exclusion criteria associated with inflammatory cytokines comprised blood cell counts or percentages, immune‐associated pathways, and immune‐related diseases (Argue et al. 2025). Additionally, we calculated the *F*‐statistics for all IVs to assess the IVs' potential power, excluding instruments with an *F*‐statistic below 10 to avoid weak instruments bias (Sanderson et al. 2022).

### 2.5 Statistical Analysis

To examine potential causal relationships between circulating cytokines and BD, we performed multiple MR methods, including inverse‐variance weighted (IVW), MR‐Egger, weighted median, simple mode, and weighted mode. The IVW method served as the primary analysis due to its statistical efficiency, though it can provide unbiased estimates only under the assumption of balanced pleiotropy. To evaluate the robustness of the results and address potential violations of this assumption, we additionally performed sensitivity analysis to assess heterogeneity and horizontal pleiotropy. MR‐Egger was performed as a sensitivity analysis to detect and account for directional pleiotropy, under the assumption that the pleiotropic effects of the instruments are independent of their associations with the exposure (InSIDE assumption), although this condition is difficult to verify, and the method is less statistically powerful than IVW. The weighted median method complements IVW, as it is robust to outliers and sensitive to valid genetic variants. The simple mode and weighted mode methods are less affected by a small number of pleiotropic genetic variants compared to IVW and MR‐Egger but may exhibit lower precision in certain scenarios (L. G. Chen et al. 2024).

In MR research, sensitivity analysis plays an essential role in assessing potential pleiotropy and heterogeneity of genetic variants. We implemented Cochran's Q statistic to investigate heterogeneity in the causal estimates of polygenic variants (*p* < 0.05 is considered as existing heterogeneity). We performed the intercept of the MR‐Egger method to examine the directional pleiotropy (*p* < 0.05 is considered as existing directional pleiotropy). Additionally, we conducted MR‐Pleiotropy RESidual Sum and Outlier methods (MR‐PRESSO) to reassess and address potential horizontal pleiotropy. After removing horizontal pleiotropy stemming from genetic variants, we applied the five methods mentioned above to investigate causal inference. Finally, we performed a leave‐one‐out analysis to estimate the dependence of the MR analysis on specific SNPs (Yu et al. 2024).

## 3 Results

### 3.1 Estimation of Causal Effects of Circulating Cytokines for Bipolar Disorder Risk

We identified independent IVs for inflammatory cytokines with ranging from 2 to 22. All IVs reached genome‐wide significance, including 41 cytokines with 218 SNPs. *F*‐statistics for these IVs ranged from 14.62 to 113.17, indicating sufficient instrument strength for MR analysis in Table S1. We excluded eight SNPs (rs9267091, rs5754733, rs7088799, rs4737732, rs396960, rs2673604, rs117509142, rs11551183) for inflammatory cytokines due to their correlations with confounders or outcomes, as identified through the Phenoscanner database in Table S2. This step ensured the validity and robustness of our analysis by addressing potential sources of bias or confounding. Additionally, a total of 17 duplicated SNPs were excluded due to their presence in multiple cytokines, which caused redundancy in Table S3.

**FIGURE 2:**
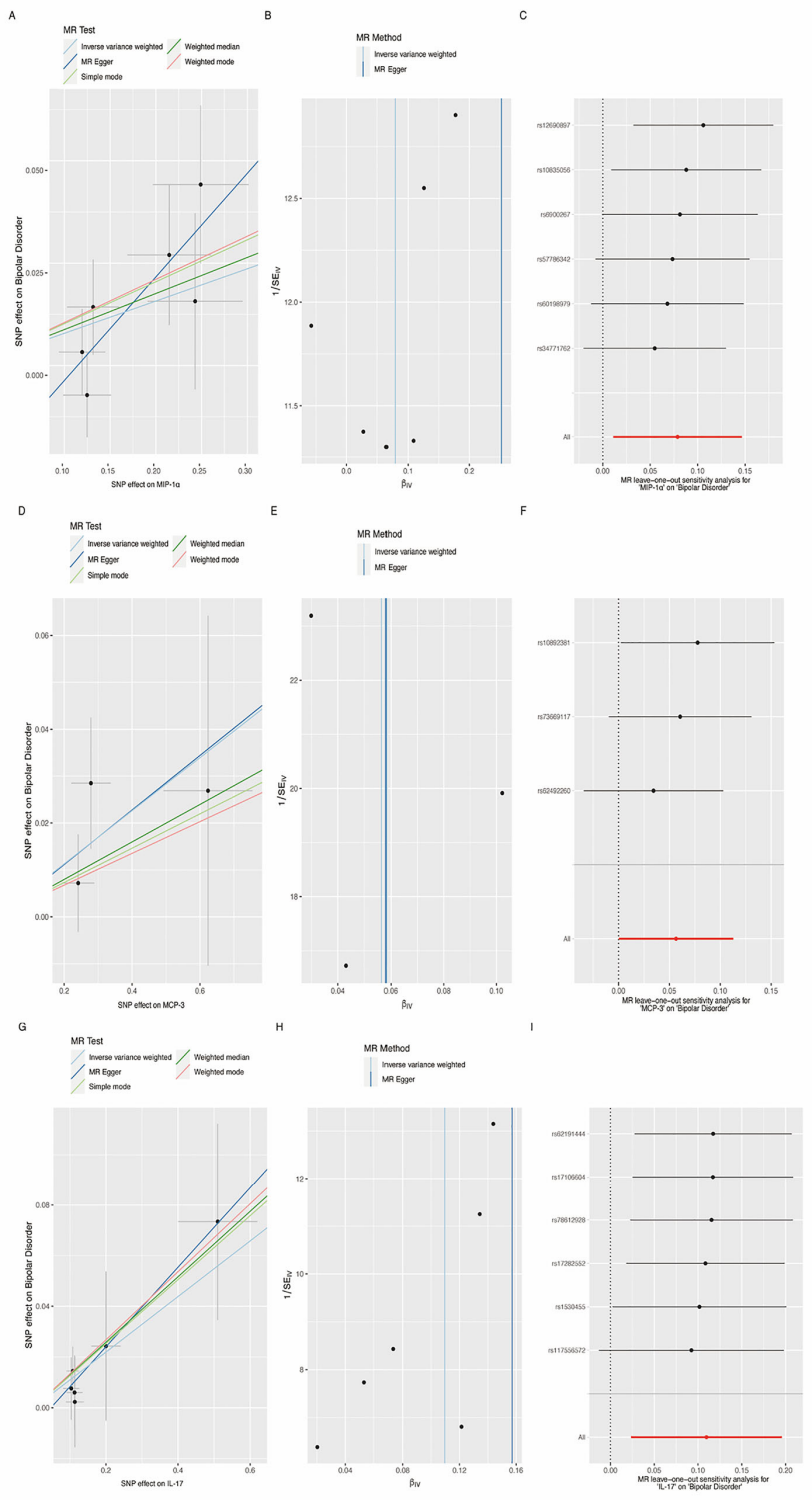
The scatter plots, funnel plots, and forest plots represented the SNPs of MIP‐1α (A–C), MCP‐3 (D–F), and IL‐17 (G–I) about circulating cytokines and the associated risk of bipolar disorder. SNPs, single nucleotide polymorphisms; MR, Mendelian randomization; MIP‐1α, macrophage inflammatory protein 1α; MCP‐3, monocyte chemotactic protein 3; IL‐17, interleukin‐17.

The genetic relationship between inflammatory cytokines and BD was depicted in Table 1 and Figure 2. Genetically driven increases in MIP‐1α and IL‐17 were associated with a higher risk of BD, as revealed by the IVW method (odds ratio [OR] = 1.084, 95% confidence interval [CI] = 1.002–1.174, *p* = 0.044; OR = 1.119, 95% CI = 1.021–1.226, *p* = 0.016) and weighted median (OR = 1.117, 95% CI = 1.011–1.234, *p* = 0.029; OR = 1.137, 95% CI = 1.021–1.266, *p* = 0.019). No statistically significant associations were observed in the MR‐Egger (OR = 1.359, 95% CI = 1.046–1.766, *p* = 0.105; OR = 1.156, 95% CI = 0.955–1.400, *p* = 0.233), simple mode (OR = 1.129, 95% CI = 0.972–1.311, *p* = 0.188; OR = 1.127, 95% CI = 0.982–1.294, *p* = 0.163), and weighted mode methods (OR = 1.131, 95% CI = 0.967–1.323, *p* = 0.198; OR = 1.141, 95% CI = 0.986–1.319, *p* = 0.150). Furthermore, genetically elevated MCP‐3 was associated with increased risk of developing BD in the IVW analysis (OR = 1.060, 95% CI = 1.001–1.122, *p* = 0.046), but not in weighted median (OR = 1.041, 95% CI = 0.971–1.116, *p* = 0.261), MR‐Egger (OR = 1.061, 95% CI = 0.837–1.346, *p* = 0.710), simple mode (OR = 1.037, 95% CI = 0.944–1.139, *p* = 0.527), and weighted mode approaches (OR = 1.034, 95% CI = 0.952–1.124, *p* = 0.511).

**TABLE 1:** Estimation of associations between circulating MIP‐1α, MCP‐3, and IL‐17 and risk of bipolar disorder.

Methods	MR results				Heterogeneity test			Horizontal pleiotropy test			
					Cochrane Q test			MR Egger intercept		MR‐PRESSO global test	
	SNP	Beta	SE	*p*	Q	df	*p*	Intercept	*p*	RSSobs	*p*
MIP‐1α											
IVW	6	0.081	0.040	0.044	4.666	4	0.323			7.405	0.378
MR Egger	6	0.307	0.133	0.105	1.562	3	0.668	−0.035	0.176		
Weighted median	6	0.111	0.051	0.029							
Simple mode	6	0.121	0.076	0.188							
Weighted mode	6	0.123	0.080	0.198							
MCP‐3											
IVW	3	0.058	0.029	0.046	1.370	2	0.504			—	—
MR Egger	3	0.059	0.121	0.710	1.370	1	0.242	−0.0004	0.992		
Weighted median	3	0.040	0.036	0.261							
Simple mode	3	0.036	0.048	0.527							
Weighted mode	3	0.034	0.042	0.511							
IL‐17											
IVW	6	0.112	0.047	0.016	0.539	4	0.970			9.031	0.397
MR Egger	6	0.145	0.097	0.233	0.393	3	0.942	−0.0052	0.728		
Weighted median	6	0.128	0.055	0.019							
Simple mode	6	0.120	0.070	0.163							
Weighted mode	6	0.132	0.074	0.150							

Abbreviations: df, degree of freedom; IL‐17, interleukin‐17; IVW, inverse variance weighted; MCP‐3, monocyte chemoattractant protein‐3; MIP‐1α, macrophage inflammatory protein‐1α; MR, mendelian randomization; MR‐PRESSO, mendelian randomization pleiotropy residual sum and outlier; RSSobs, observed residual sum of squares; SNP, single‐nucleotide polymorphism; SE, standard error.

Sensitivity analyses using Cochran's Q test from the IVW and MR‐Egger analysis demonstrated no significant heterogeneity in the causal effects of MIP‐1α, MCP‐3, and IL‐17 on BD shown in Table 1 and Figure 2 (all *p*‐values > 0.05). Additionally, the intercept from the MR‐Egger approach suggested no directional pleiotropy for these cytokines shown in Table 1 (intercept = −0.035, *p* = 0.176; intercept = −0.0004, *p* = 0.992; intercept = −0.0052, *p* = 0.728, respectively). Finally, leave‐one‐out sensitivity analysis identified no individual SNP with a strong effect on the overall influence displayed in Figure 2. These robust findings support cytokine‐mediated genetic risk for BD from MIP‐1α, MCP‐3, and IL‐17.

### 3.2 Estimation of Causal Effects of Bipolar Disorder for Blood Cytokines Risk

In our study on BD, a total of 64 independent genome‐wide significant SNPs were utilized as IVs. However, after conducting Phenoscanner research, 10 SNPs (rs6992333, rs4619651, rs3088186, rs2336147, rs174592, rs13195402, rs13044225, rs12289486, rs11870683, rs112481526) were excluded from this study owing to their associations with the confounders or the outcomes, to ensure the validity of our analysis in Table S4. And after excluding the palindromic sequence SNPs, we finally obtained a total of 53 SNPs in the MR analysis of the causal effect of BD on blood cytokines in Table S5.

**FIGURE 3:**
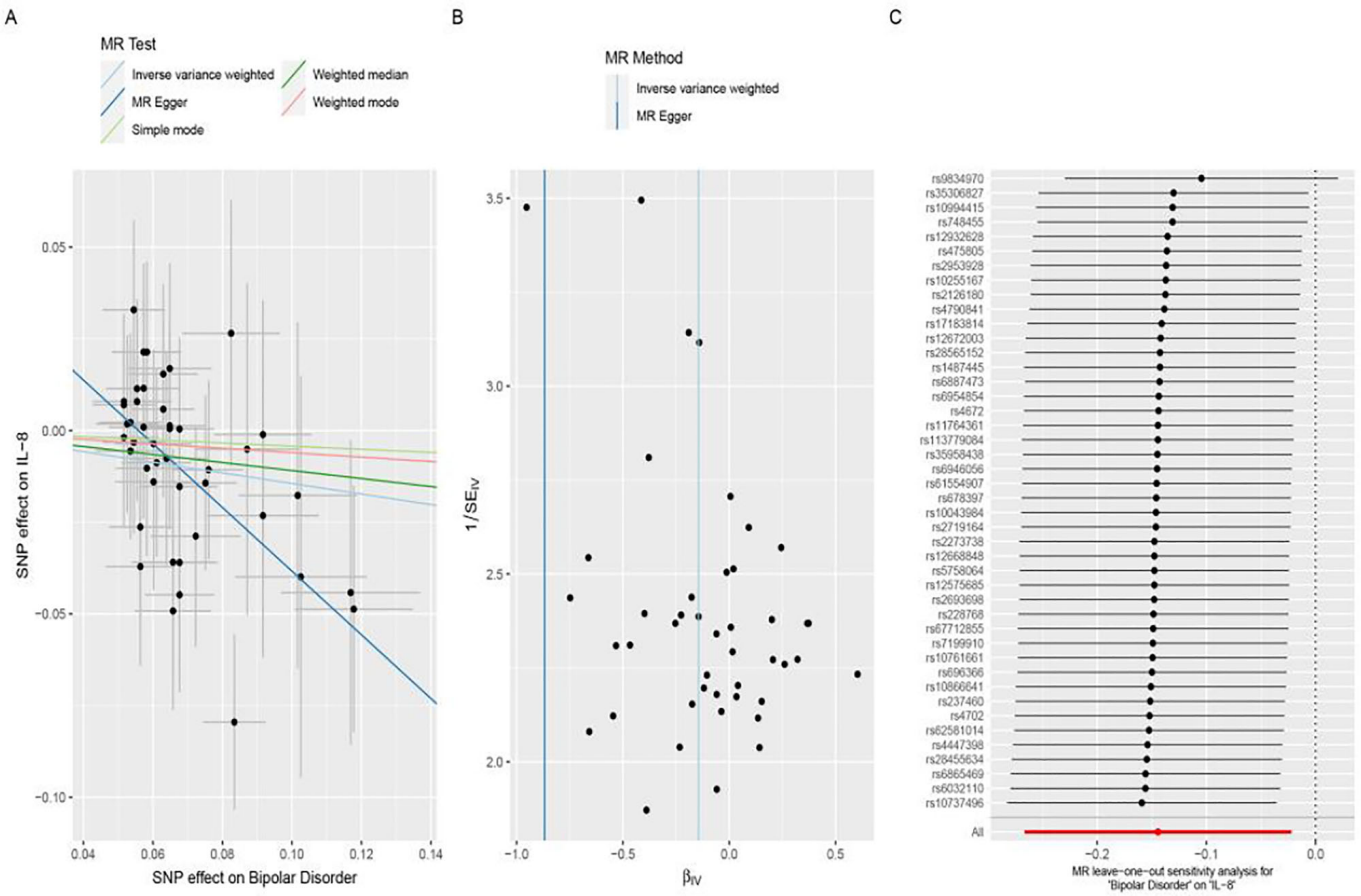
The scatter plots, funnel plots, and forest plots represented the SNPs of IL‐8 (A–C) from bipolar disorder and the associated risk of circulating cytokines. SNPs, single nucleotide polymorphisms; MR, Mendelian randomization; IL‐8, interleukin‐8.

The results found that genetic liability to BD was protective of IL‐8, as inferred from the IVW (OR = 0.866, 95% CI = 0.766–0.978, *p* = 0.021) and MR‐Egger methods (OR = 0.420, 95% CI = 0.230–0.765, *p* = 0.007) as shown in Table 2 and Figure 3. However, inconsistent results were observed when using the weighted median (OR = 0.897, 95% CI = 0.756–1.064, *p* = 0.211), simple mode (OR = 0.958, 95% CI = 0.646–1.422, *p* = 0.833), and weighted mode approaches (OR = 0.941, 95% CI = 0.657–1.349, *p* = 0.743). The distinct results of these five MR approaches demonstrate that the causal effects of genetic liability to BD on IL‐8 are suggestive. Besides, MR‐Egger intercept analysis revealed the presence of horizontal pleiotropy, as the analysis of BD variants associated with IL‐8 was not statistically significant, as shown in Table 2 and Figure 3 (intercept = 0.048, *p* = 0.020). Given this methodological heterogeneity and evidence of pleiotropy, the putative protective effect of BD genetic liability on IL‐8 levels should be interpreted with caution.

**TABLE 2:** Estimation of associations between bipolar disorder and risk of circulating levels of IL‐8.

Methods	MR results				Heterogeneity test			Horizontal pleiotropy test			
					Cochrane Q test			MR Egger intercept		MR‐PRESSO global test	
	SNP	Beta	SE	*p*	Q	df	*p*	Intercept	*p*	RSSobs	*p*
IL‐8											
IVW	44	−0.144	0.062	0.021	29.792	43	0.937			42.050	0.880
MR Egger	44	−0.868	0.307	0.007	23.977	42	0.989	0.048	0.020		
Weighted median	44	−0.109	0.087	0.211							
Simple mode	44	−0.043	0.201	0.833							
Weighted mode	44	−0.060	0.184	0.743							

Abbreviations: df, degree of freedom; IL‐8, Interleukin‐8; IVW, inverse variance weighted; MR, Mendelian randomization; MR‐PRESSO, Mendelian randomization pleiotropy residual sum and outlier; RSSobs, observed residual sum of squares; SE, standard error; SNP, single‐nucleotide polymorphism.

## 4 Discussion

Leveraging MR analysis as a robust statistical technique that minimizes confounding factors, we can thus estimate causal effects (Sanderson et al. 2022). In this study, three peripheral inflammatory cytokines, namely IL‐17, MIP‐1α, and MCP‐3, were observed to have causal effects on BD, thereby increasing susceptibility to developing the disorder. However, we found only weak evidence for a protective effect of BD on IL‐8 levels, which failed to withstand sensitivity analysis. Taken together, our findings support the role of inflammatory cytokines as risk factors for BD, contributing to a comprehensive understanding of the inflammation hypothesis in BD pathogenesis.

Overall, these inflammatory cytokines may influence the risk of BD. The influence of inflammatory cytokines on BD is indeed multifaceted, encompassing dynamic interactions among neuroimmune signaling, neurotransmitter systems, and hypothalamic—pituitary–adrenal (HPA) axis alterations—all of which contribute to structural and functional brain changes. In individuals with BD, activated microglia and increased levels of proinflammatory cytokines have been observed to drive neuroinflammation, leading to blood–brain barrier dysfunction, excessive synaptic pruning, and oxidative stress, which can contribute to mood symptoms (Argue et al. 2025; Bacchi 2025). Furthermore, imbalances in monoaminergic and glutamatergic systems, along with HPA axis overactivation in response to the inflammatory state, were also reported during the development and course of BD (Almeida et al. 2020; Kadriu et al. 2021). In addition, a recent study has revealed that inflammation and immune activation play a role in disrupting white matter integrity in BD, thereby affecting clinical symptoms (Cao et al. 2025). These results show that neuroinflammatory changes in BD may be more complex than previously thought, and more studies are required to deepen our understanding of inflammatory mechanisms in the pathophysiology of BD.

Previous research has revealed causality between immune cells and BD (Y. Zhang, Wang, et al. 2024). To our knowledge, this study is the first to identify a causative link between immune cytokines and BD, with increased IL‐17 levels associated with a high risk of BD. IL‐17 is a pro‐inflammatory cytokine released by T helper 17 (Th17) cells, inducing other cytokines and chemokines to promote inflammation. Regulatory T (Treg) cells can inhibit Th17 cells proliferation and reduce IL‐17 secretion to suppress immune response with anti‐inflammatory effects. Accumulating research has shown that IL‐17 can modulate inflammation in the central nervous system (CNS), involving endothelial cells, microglia, astrocytes, and neurons (Beurel et al. 2022). Compared to the general population, IL‐17 level in blood are higher in individuals with BD (Wu et al. 2022); an imbalance of Th17 and Treg cells has been observed in BD patients, with increased Th17 cell percentages and decreased Treg cell numbers (G. Chen et al. 2023). Besides, IL‐17 levels have been found to fluctuate during different phases of BD, with lower levels in depressive episodes and higher levels in mania episodes (Wu et al. 2022), suggesting that alteration of IL‐17 in BD patients may be state‐dependent. Furthermore, serum IL‐17 levels were positively correlated with duration of BD (Keshri et al. 2018); a downward trend in plasm IL‐17 levels has also been associated with positive therapeutic responses in manic BD patients following treatment (H.Z. Li et al. 2015), demonstrating that IL‐17 expression may serve as a potential neurobiological staging marker involved in progression of BD. Finally, biologic therapies, such as brodalumab and ixekizumab (anti‐IL‐17 antibodies), have been proven effective with antidepressant effects (Hassamal 2023). Similarly, monoclonal antibodies targeting IL‐17 ameliorated depression and reduced suicidal ideation and behavior in psoriasis patients with increased serum IL‐17 levels, who were more prone to comorbid depression and suicidality compared to healthy individuals (Schiweck et al. 2023). In line with this, anti‐IL‐17 treatment also alleviated anxiety‐ and depression‐like behavior in mice (Kunisawa et al. 2022). In accordance with our present findings, these studies support a causal role for IL‐17 in BD. However, a previous MR study did not find a causal relationship between IL‐17 and BD (Perry et al. 2021). These contradictions can be explained by several reasons. Above all, differences in filtering criteria of IVs resulted in varying numbers of SNPs, which contributed to the discrepancies in findings. In addition, the constrained focus on coding‐region SNPs (cis‐variants) in prior studies led to diminished variant sets for analysis, which failed to yield evidence supporting the significant associations.

Our findings demonstrated a causal relationship of MIP‐1α with BD. MIP‐1α, also known as C–C motif chemokine 3 (CCL3), is a neutrophil chemoattractant with pro‐inflammatory effects in immune system regulation. MIP‐1α is essential for maintaining homeostasis through crosstalk between neurons and glial cells during central CNS inflammation (Sowa and Tokarski 2021). Serum MIP‐1α levels showed no significant differences between patients with BD and healthy individuals. However, elevated serum levels of MIP‐1α were associated with depression (Harsanyi et al. 2022). In addition, a postmortem study of cortex tissue found that increased MIP‐1α expression in glia cells of patients with manic depressive illness (Ishizuka et al. 1997). One explanation for this divergence could be the heterogeneity and complexity of BD, including different phases and sample size variations. Finally, other studies found that IL‐17 can induce MIP‐1a expression in a mouse model (Y. Zhang et al. 2016). It is speculated that elevated IL‐17 and MIP‐1a may collectively regulate neuroinflammation pathways implicated in the development of BD. Future studies on their potential interaction as inflammatory biomarkers across different phases of BD are warranted.

We observed that MCP‐3 was associated with increased risk of developing BD. MCP‐3, called CCL7, is a chemotactic factor that can induce the migration of monocyte‐derived macrophages to inflamed or injured tissues or organs during immune responses. In CNS, MCP‐3 secretion from astrocytes facilitates microglia activation and acts as a pro‐inflammatory mediator (Xue et al. 2021). Previous research uncovered aberrant MCP‐3 expression in patients with BD, with this expression positively correlated with manic symptoms and illness duration (Haarman et al. 2014). What's more, MCP‐3 expression was significantly upregulated by IL‐17 treatment (Daisuke et al. 2019). These findings support the IL‐17 network as a potential neurobiological marker in BD pathogenesis. Prior research has found that IL‐17 signaling pathways play a crucial part in mental disorders, especially in depression (Bliźniewska‐Kowalska et al. 2023). Furthermore, certain inflammatory markers, such as IL‐17 signaling pathways, have been recognized to discriminate between different subtypes of mood disorder (Xu et al. 2024). Therefore, the involvement of IL‐17 and its signaling pathways in the pathogenesis of BD, along with the interactions among various cytokines, deserves further investigation.

## 5 Limitations

Our study has several limitations that warrant consideration. First, the GWAS summary‐level data used in this study were predominantly derived from European populations, which may limit the generalizability of our findings to other ethnicities. Genetic variations and their associations between blood cytokines and BD could differ across diverse populations. Therefore, caution should be exercised when extrapolating our results to non‐European populations, and future studies including a more diverse range of participants are needed to validate these findings across ethnicities. Second, the absence of individual‐level information in the GWAS summary data hinders our ability to investigate population stratification. Population stratification refers to systematic differences in allele frequency across subpopulations within the BD cohort, which can introduce analytical bias. Without individual‐level data, we cannot account for potential confounding factors related to population stratification. In addition, subtypes and episodes of BD were not further analyzed due to data constraints. Lastly, while we conducted robust sensitivity analyses to detect and address potential horizontal pleiotropic effects, complete elimination of such effects remains challenging. Horizontal pleiotropy occurs when genetic variants influence both the exposure (inflammatory cytokines) and the outcome (BD) through independent pathways, leading to bias in causal estimates. Although we employed various methods to assess and mitigate pleiotropy, unmeasured or residual pleiotropic effects cannot be completely ruled out. These limitations underscore the need for further research in diverse populations, using individual‐level data and advanced methods to address potential biases and account for pleiotropy, thereby enhancing our comprehensive understanding of the causal relationship between circulating inflammatory cytokines and BD.

## 6 Conclusion

In summary, this study demonstrates that genetically determined variations in circulating cytokines‐including IL‐17, MIP‐1α, and MCP‐3‐increase the risk of BD. These identified immunoregulatory factors may act as upstream roles in BD etiology and pathogenesis, serving as potential biomarkers for early diagnosis and intervention of BD. Our findings deepen the understanding of BD's underlying inflammatory mechanisms and provide insights into potential clinical management of BD.

## Author Contributions

**Ying Cheng**: writing – original draft, writing – review and editing. **Meiti Wang**: writing – original draft, writing – review and editing. **Yu Fang**: data curation, visualization. **Jinjie Xu**: data curation, visualization. **Jinxin Zheng**: formal analysis, methodology. **Peijun Ju**: conceptualization, supervision, writing, review, and editing. **Jianhua Chen**: conceptualization, supervision, funding acquisition, writing – review and editing.

## Conflicts of Interest

The authors declare no conflicts of interest.

## Peer Review

The peer review history for this article is available at https://publons.com/publon/10.1002/brb3.70796.

## Ethics Statement

Ethical approval was not provided for this study on human participants because we used the publicly available GWAS catalog to conduct a two‐sample MR study. No additional ethical approval was required due to the re‐analysis of previously summarized data.

## Supporting information




**Supplementary Tables**: brb370796‐sup‐0001‐tableS1‐S5.xlsx

## Data Availability

This research was implemented using the publicly available genome‐wide association studies (GWAS) database resource.

## References

[brb370796-bib-0001] Ahola‐Olli, A. V. , P. Wurtz , A. S. Havulinna , et al. 2017. “Genome‐Wide Association Study Identifies 27 Loci Influencing Concentrations of Circulating Cytokines and Growth Factors.” American Journal of Human Genetics 100, no. 1: 40–50. 10.1016/j.ajhg.2016.11.007. 27989323 PMC5223028

[brb370796-bib-0002] Almeida, P. G. C. , J. V. Nani , J. P. Oses , E. Brietzke , and M. A. F. Hayashi . 2020. “Neuroinflammation and Glial Cell Activation in Mental Disorders.” Brain, Behavior, & Immunity ‐ Health 2: 100034. 10.1016/j.bbih.2019.100034. PMC8474594 38377429

[brb370796-bib-0003] Argue, B. M. R. , L. G. Casten , S. McCool , et al. 2025. “Immune Dysregulation in Bipolar Disorder.” Journal of Affective Disorders 374: 587–597. 10.1016/j.jad.2025.01.062. 39818340 PMC11830520

[brb370796-bib-0004] Bacchi, A. D. 2025. “Beyond the Neuron: The Integrated Role of Glia in Psychiatric Disorders.” Neuroglia 6, no. 2: 15. 10.3390/neuroglia6020015.

[brb370796-bib-0005] Beurel, E. , E. M. Medina‐Rodriguez , and R. S. Jope . 2022. “Targeting the Adaptive Immune System in Depression: Focus on T Helper 17 Cells.” Pharmacological Reviews 74, no. 2: 373–386. 10.1124/pharmrev.120.000256. 35302045 PMC8973514

[brb370796-bib-0006] Bliźniewska‐Kowalska, K. , A. Halaris , P. Gałecki , and M. Gałecka . 2023. “Role of Interleukin 17 (IL‐17) in the Inflammatory Hypothesis of Depression.” Journal of Affective Disorders Reports 14: 100610. 10.1016/j.jadr.2023.100610.

[brb370796-bib-0007] Burgess, S. , A. Butterworth , and S. G. Thompson . 2013. “Mendelian Randomization Analysis With Multiple Genetic Variants Using Summarized Data.” Genetic Epidemiology 37, no. 7: 658–665. 10.1002/gepi.21758. 24114802 PMC4377079

[brb370796-bib-0008] Cao, Y. , P. Lizano , M. Li , et al. 2025. “White Matter Microstructural and Inflammation‐based Subgroups in Bipolar Disorder II Depression Differentiate in Depressive and Psychotic Symptoms.” Journal of Affective Disorders 368: 493–502. 10.1016/j.jad.2024.09.112. 39299597

[brb370796-bib-0009] Chen, L. G. , J. D. Tubbs , Z. Liu , T. Q. Thach , and P. C. Sham . 2024. “Mendelian Randomization: Causal Inference Leveraging Genetic Data.” Psychological Medicine 54, no. 8: 1461–1474. 10.1017/s0033291724000321. 38639006

[brb370796-bib-0010] Chen, Z. , Y. Huang , B. Wang , et al. 2023. “T Cells: An Emerging Cast of Roles in Bipolar Disorder.” Transl Psychiatry 13, no. 1: 153. 10.1038/s41398-023-02445-y. 37156764 PMC10167236

[brb370796-bib-0011] Daisuke, I. , H. Toshio , and O. Naomi . 2019. “Effect of IL‐17 for Monocyte Chemotactic Protein Production by Human Temporomandibular Joint Synovial Fibroblasts.” International Journal of Oral‐Medical Sciences 18, no. 1: 1–9. 10.5466/ijoms.18.1.

[brb370796-bib-0012] Dembek, C. , D. Mackie , K. Modi , Y. Zhu , X. Niu , and T. Grinnell . 2023. “The Economic and Humanistic Burden of Bipolar Disorder in Adults in the United States.” Annals of General Psychiatry 22, no. 1: 13. 10.1186/s12991-023-00440-7. 36964564 PMC10037816

[brb370796-bib-0013] Firth, J. , M. Solmi , R. E. Wootton , et al. 2020. “A Meta‐Review of ‘Lifestyle Psychiatry’: The Role of Exercise, Smoking, Diet and Sleep in the Prevention and Treatment of Mental Disorders.” World Psychiatry 19, no. 3: 360–380. 10.1002/wps.20773. 32931092 PMC7491615

[brb370796-bib-0014] Cross‐Disorder Group of the Psychiatric Genomics Consortium . 2019. “Genomic Relationships, Novel Loci, and Pleiotropic Mechanisms across Eight Psychiatric Disorders.” Cell 179, no. 7: 1469–1482.e1411. 10.1016/j.cell.2019.11.020. 31835028 PMC7077032

[brb370796-bib-0015] Haarman, B. C. , R. F. Riemersma‐Van der Lek , H. Burger , et al. 2014. “Relationship between Clinical Features and Inflammation‐Related Monocyte Gene Expression in Bipolar Disorder–Towards a Better Understanding of Psychoimmunological Interactions.” Bipolar Disorders 16, no. 2: 137–150. 10.1111/bdi.12142. 24286609

[brb370796-bib-0016] Harsanyi, S. , I. Kupcova , L. Danisovic , and M. Klein . 2022. “Selected Biomarkers of Depression: What Are the Effects of Cytokines and Inflammation?” International Journal of Molecular Sciences 24, no. 1: 578. 10.3390/ijms24010578. 36614020 PMC9820159

[brb370796-bib-0017] Hassamal, S. 2023. “Chronic Stress, Neuroinflammation, and Depression: An Overview of Pathophysiological Mechanisms and Emerging Anti‐inflammatories.” Frontiers in Psychiatry 14: 1130989. 10.3389/fpsyt.2023.1130989. 37252156 PMC10213648

[brb370796-bib-0018] Ishizuka, K. , R. Igata‐Yi , T. Kimura , et al. 1997. “Expression and Distribution of CC Chemokine Macrophage Inflammatory Protein‐1 Alpha/LD78 in the human Brain.” Neuroreport 8, no. 5: 1215–1218. 10.1097/00001756-199703240-00031. 9175116

[brb370796-bib-0019] Kadriu, B. , C. A. Farmer , P. Yuan , et al. 2021. “The Kynurenine Pathway and Bipolar Disorder: Intersection of the Monoaminergic and Glutamatergic Systems and Immune Response.” Molecular Psychiatry 26, no. 8: 4085–4095. 10.1038/s41380-019-0589-8. 31732715 PMC7225078

[brb370796-bib-0020] Keshri, N. , H. Nandeesha , and S. Kattimani . 2018. “Elevated Interleukin‐17 and Reduced Testosterone in Bipolar Disorder. Relation with Suicidal Behaviour.” Asian Journal of Psychiatry 36: 66–68. 10.1016/j.ajp.2018.06.011. 29979995

[brb370796-bib-0021] Kunisawa, K. , J. Shan , Q. Lu , et al. 2022. “Loureirin C and Xanthoceraside Attenuate Depression‐Like Behaviors and Expression of Interleukin‐17 in the Prefrontal Cortex Induced by Chronic Unpredictable Mild Stress in Mice.” Neurochemical Research 47, no. 9: 2880–2889. 10.1007/s11064-022-03692-z. 35871434

[brb370796-bib-0022] Li, G. , Q. He , M. Sun , et al. 2024. “Association of Healthy Lifestyle Factors and Genetic Liability with Bipolar Disorder: Findings From the UK Biobank.” Journal of Affective Disorders 364: 279–285. 10.1016/j.jad.2024.08.011. 39137837

[brb370796-bib-0023] Li, H. Z. , W. Hong , C. Zhang , et al. 2015. “IL‐23 and TGF‐β1 Levels as Potential Predictive Biomarkers in Treatment of Bipolar I Disorder With Acute Manic Episode.” Journal of Affective Disorders 174: 361–366. 10.1016/j.jad.2014.12.033. 25545602

[brb370796-bib-0024] McIntyre, R. S. , M. Berk , E. Brietzke , et al. 2020. “Bipolar Disorders.” Lancet 396, no. 10265: 1841–1856. 10.1016/S0140-6736(20)31544-0. 33278937

[brb370796-bib-0025] Mullins, N. , A. J. Forstner , K. S. O'Connell , et al. 2021. “Genome‐Wide Association Study of More than 40,000 Bipolar Disorder Cases Provides New Insights Into the Underlying Biology.” Nature Genetics 53, no. 6: 817–+. 10.1038/s41588-021-00857-4. 34002096 PMC8192451

[brb370796-bib-0026] Pape, K. , R. Tamouza , M. Leboyer , and F. Zipp . 2019. “Immunoneuropsychiatry—Novel Perspectives on Brain Disorders.” Nature Reviews Neurology 15, no. 6: 317–328. 30988501 10.1038/s41582-019-0174-4

[brb370796-bib-0027] Perry, B. I. , R. Upthegrove , N. Kappelmann , P. B. Jones , S. Burgess , and G. M. Khandaker . 2021. “Associations of Immunological Proteins/Traits With Schizophrenia, Major Depression and Bipolar Disorder: A Bi‐Directional Two‐Sample Mendelian Randomization Study.” Brain, Behavior, and Immunity 97: 176–185. 10.1016/j.bbi.2021.07.009. 34280516 PMC7612947

[brb370796-bib-0028] Poletti, S. , M. G. Mazza , and F. Benedetti . 2024. “Inflammatory Mediators in Major Depression and Bipolar Disorder.” Translational Psychiatry 14, no. 1: 247. 10.1038/s41398-024-02921-z. 38851764 PMC11162479

[brb370796-bib-0029] Ruiz‐Sastre, P. , C. Gómez‐Sánchez‐Lafuente , J. Martín‐Martín , et al. 2024. “Pharmacotherapeutic Value of Inflammatory and Neurotrophic Biomarkers in Bipolar Disorder: A Systematic Review.” Progress in Neuro‐Psychopharmacology & Biological Psychiatry 134: 111056. 10.1016/j.pnpbp.2024.111056. 38879067

[brb370796-bib-0030] Saccaro, L. F. , J. Crokaert , N. Perroud , and C. Piguet . 2023. “Structural and Functional MRI Correlates of Inflammation in Bipolar Disorder: A Systematic Review.” Journal of Affective Disorders 325: 83–92. 10.1016/j.jad.2022.12.162. 36621677

[brb370796-bib-0031] Sanderson, E. , M. M. Glymour , M. V. Holmes , et al. 2022. “Mendelian Randomization.” Nature Reviews Methods Primers 2, no. 1: 6. 10.1038/s43586-021-00092-5. PMC7614635 37325194

[brb370796-bib-0032] Schiweck, C. , M. Aichholzer , A. Reif , and S. E. Thanarajah . 2023. “Targeting IL‐17A Signaling in Suicidality, Promise or the Long Arm of Coincidence? Evidence in Psychiatric Populations Revisited.” Journal of Affective Disorders Reports 11: 100454. 10.1016/j.jadr.2022.100454.

[brb370796-bib-0033] Sowa, J. E. , and K. Tokarski . 2021. “Cellular, Synaptic, and Network Effects of Chemokines in the Central Nervous System and Their Implications to Behavior.” Pharmacological Reports 73, no. 6: 1595–1625. 10.1007/s43440-021-00323-2. 34498203 PMC8599319

[brb370796-bib-0034] Wu, X. , Z. Chen , Y. Liao , et al. 2022. “Are Serum Levels of Inflammatory Markers Associated With the Severity of Symptoms of Bipolar Disorder?” Frontiers in Psychiatry 13: 1063479. 10.3389/fpsyt.2022.1063479. 36741577 PMC9894870

[brb370796-bib-0035] Xu, F. , Y. Su , X. Wang , T. Zhang , T. Xie , and Y. Wang . 2024. “Olink Proteomics Analysis Uncovers Inflammatory Proteins in Patients With Different States of Bipolar Disorder.” International Immunopharmacology 131: 111816. 38484669 10.1016/j.intimp.2024.111816

[brb370796-bib-0036] Xue, J. , Y. Zhang , J. Zhang , Z. Zhu , Q. Lv , and J. Su . 2021. “Astrocyte‐Derived CCL7 Promotes Microglia‐Mediated Inflammation Following Traumatic Brain Injury.” International Immunopharmacology 99: 107975. 10.1016/j.intimp.2021.107975. 34293712

[brb370796-bib-0037] Yu, T. , C. Chen , Y. Yang , et al. 2024. “Dissecting the Association Between Gut Microbiota, Body Mass Index and Specific Depressive Symptoms: A Mediation Mendelian Randomisation Study.” General Psychiatry 37, no. 4: e101412. 10.1136/gpsych-2023-101412. 38975363 PMC11227829

[brb370796-bib-0038] Zhang, J. , K. Li , and X. Qiu . 2024. “Exploring Causal Correlations Between Inflammatory Cytokines and Knee Osteoarthritis: A Two‐Sample Mendelian Randomization.” Frontiers in Immunology 15: 1362012. 10.3389/fimmu.2024.1362012. 38698846 PMC11063282

[brb370796-bib-0039] Zhang, Y. , R. Huang , Y. Zhang , et al. 2016. “IL‐17 Induces MIP‐1alpha Expression in Primary Mouse Astrocytes via TRPC Channel.” Inflammopharmacology 24, no. 1: 33–42. 10.1007/s10787-015-0256-x. 26782821

[brb370796-bib-0040] Zhang, Y. , S.‐W. Wang , J. Ding , et al. 2024. “Causal Role of Immune Cells in Major Depressive Disorder and Bipolar Disorder: Mendelian Randomization (MR) Study.” Journal of Affective Disorders 361: 165–171. 10.1016/j.jad.2024.05.106. 38838789

